# Primitive neuroectodermal tumor of the cervix: a case report

**DOI:** 10.1186/1752-1947-5-489

**Published:** 2011-09-30

**Authors:** Farah Farzaneh, Hamidreza Rezvani, Parisa Taherzadeh Boroujeni, Farzaneh Rahimi

**Affiliations:** 1 Infertility and Reproductive Health Research Center, Department of Obstetrics & Gynecology, Taleghani Hospital, Tehran, Iran; 2 Department of Medical Oncology, Taleghani Hospital, Tehran, Iran; 3 Department of Obstetrics & Gynecology, Taleghani Hospital, Tehran, Iran; 4 Department of Cytopathology, Taleghani Hospital, Tehran, Iran

## Abstract

**Introduction:**

Peripheral primitive neuroectodermal tumor of the cervix uteri is extremely rare. Between 1987 and 2010, there were only nine cases reported in the English literature, with considerably different management policies.

**Case presentation:**

A 45-year-old Iranian woman presented to our facility with a primitive neuroectodermal tumor of the cervix uteri. Her clinical stage IB2 tumor was treated successfully with chemotherapy. Our patient underwent radical hysterectomy. There was no trace of the tumor after four years of follow-up.

**Conclusions:**

According to current knowledge, primitive neuroectodermal tumors belong to the Ewing's sarcoma family, and the improvement of treatment outcome in our patient was due to dose-intensive neoadjuvant chemotherapy, surgery and consolidation chemotherapy in accordance with the protocol for bony Ewing's sarcoma.

## Introduction

Peripheral primitive neuroectodermal tumor (PNET) of the cervix is extremely rare, and to the best of our knowledge between 1987 and 2010 only nine cases were described in the English literature [1-8]. The protocol for treatment in these previous cases varied considerably, partly due to the rarity of the disease and partly due to the different time periods of diagnosis and treatment.

## Case presentation

A 45-year-old Iranian woman presented to our University Hospital with a PNET of the cervix. After clinical staging and discussion at our Gynecology Oncology Multidisciplinary Team (GOMDT) meeting, treatment began.

Our patient was multiparous and had initially presented to a local regional facility with yellow purulent vaginal discharge for the last three months. Upon examination by a gynecologist, a biopsy was taken from a bulging cervical tumor, and a diagnosis of a small round cell malignant tumor was made based on analysis of the sample. Our patient was then referred to our center. Bimanual pelvic examination under general anesthesia revealed a 4 × 5 cm mass apparently arising from the anterior lip of the cervix, producing yellow vaginal discharge; the size of the uterus was around the size of a 10 week pregnancy. There was no extension of the lesion into the vagina, parametria, or adjacent organs including the bladder and rectum. The tumor was clinically at stage IB2. A repeat cervical biopsy was taken for confirmation of the tumor type, which was prepared and analyzed by an expert cytopathologist using immunohistochemistry (IHC).

The slides of the biopsy taken in the regional hospital were revised, and additional tumor material from the second biopsy taken in our institute was examined. Both biopsies showed the same histological appearances: small blue-staining tumor cells with little cytoplasm lying closely packed in sheets without rosette or gland formation. The cytoplasm of the tumor cells was clearly shown to contain glycogen on staining with periodic acid-Schiff (PAS). Immunohistochemistry stains for a number of epithelial markers were negative including CD3, terminal deoxynucleotidyl transferase (TdT), desmin, latent class analysis (LCA), neurofilament, CD10, CD20, cytokeratin, and carcinoembryonic antigen (CEA). However CD99, chromogranin, and synaptophysin showed strong positivity and neuron-specific enolase (NSE) was focally positive. On the basis of these findings, a diagnosis of PNET of the cervix was made.

Laboratory examination results from hematology, electrolyte, liver and renal function tests were normal. Spiral computerized tomography (CT) scanning of the chest and abdomen showed multiple para-aortic adenopathies (20 mm in diameter). The results of rectosigmoidoscopy, as well as a whole body scan, were normal. There were no signs of lung or liver metastasis.

After discussion in our GOMDT meeting, it was decided to treat our patient in the following manner: (1) Initially, our patient would received a neoadjuvant chemotherapy regimen consisting of vincristine 2 g, adriamycin 75 mg/m^2^, cyclophosphamide 1200 mg/m^2 ^(VAC) alternating with ifosfamide 1800 mg/m^2 ^plus etoposide 100 mg/m^2 ^during days 1 to 5; (2) then, surgical treatment would be performed consisting of hysterectomy and bilateral oophorectomy with or without lymphadenectomy depending on the findings at surgery; (3) finally, chemotherapy or radiotherapy depending on the findings at surgery and microscopic examination would be started. A decision regarding consolidation therapy would then ensue.

At the end of 12 weeks of chemotherapy, there was a complete response of the primary tumor, and enlarged para-aortic lymph nodes were revealed on CT scan. A radical hysterectomy was therefore performed involving the uterus and bilateral ovaries along with the proximal third of the vagina, and the bilateral parametrium, which were all removed. At the time of surgery there were no enlarged pelvic or para-aortic lymph nodes. As the para-aortic lymph nodes were not biopsied before chemotherapy due to our patient's condition, we decided to perform a complete para-aortic and pelvic lymphadenectomy in addition to a radical hysterectomy. Unfortunately, our patient's general condition prohibited a complete para-aortic and pelvic lymphadenectomy, so only sampling of the pelvic nodes was performed.

**Figure 1:**
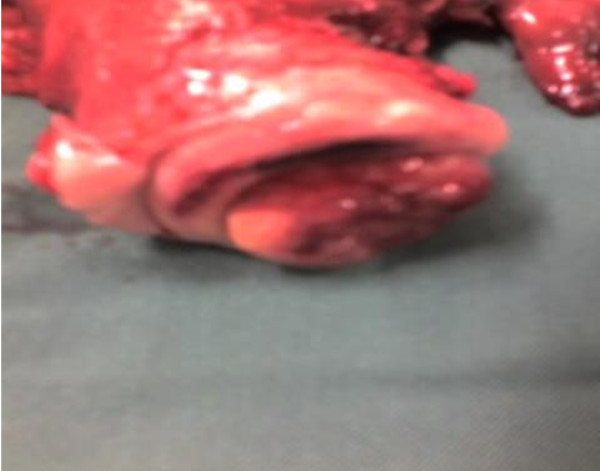
**Macroscopic view of the resected uterus**.

**Figure 2:**
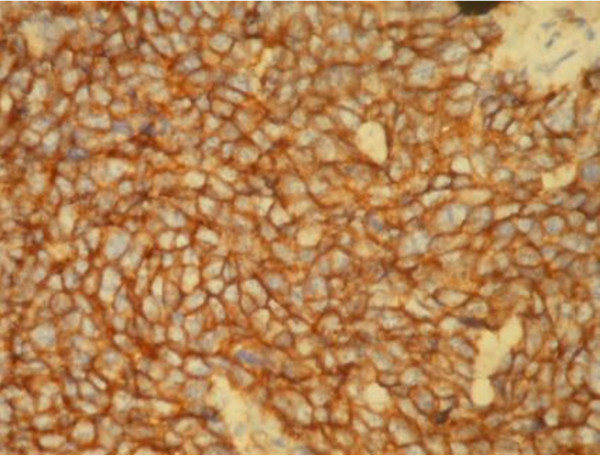
**Positive MIC2 (CD99) expression in the tumor cells**.

**Figure 3:**
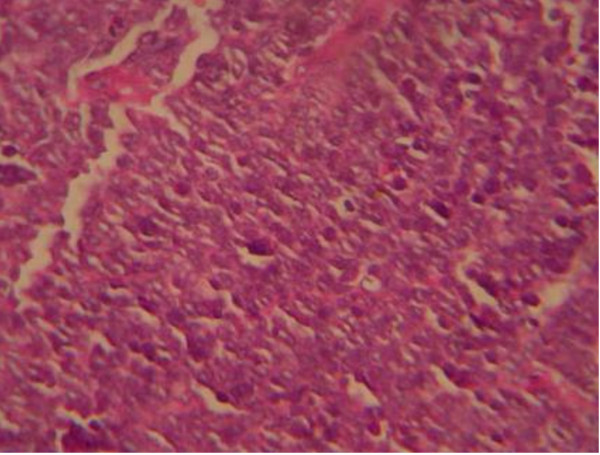
**Representative hematoxylin and eosin staining of the tumor**.

**Figure 4:**
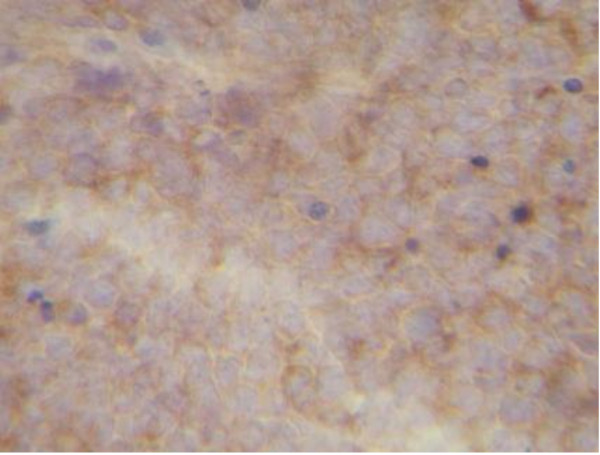
**Positive synaptophysin stain in the tumor**.

**Figure 5:**
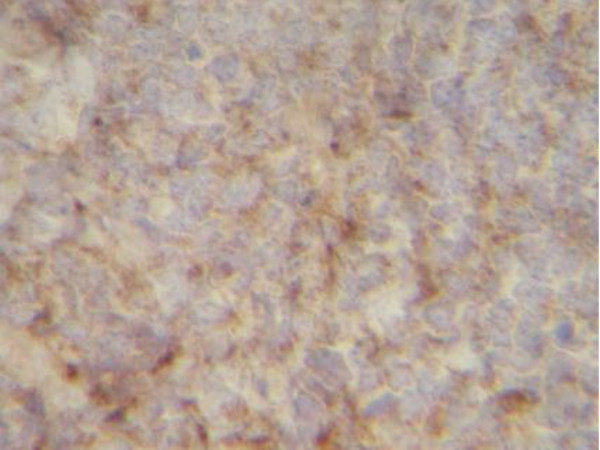
**Tumor cells shown to be chromogranin A positive**.

A macroscopic examination showed a tumor of 5 × 6 × 5 cm in the cervical location (Figure 1). Microscopic examination of the removed tumor tissue showed a small round cell tumor of the cervix with a depth of 8 mm to the stroma. In the histological examination of uterus, vagina and parametrium no malignancy was reported. No lymphovascular invasion was reported. Only three pelvic nodes from the pelvic left side and right pelvic nodes were found along with the specimen, which was negative for malignancy. All the pathology reports were reviewed by another expert pathologist (Figures 2, 3, 4, 5).

After surgery, the same chemotherapy protocol was continued for another 12 weeks. Finally, after completion of the treatment, chest and abdomen CT scans consistently showed no enlargement of lymph nodes and no sign of tumor.

Over more than four years of follow-up in our out-patient clinic (every month for three months, then every three months for the first year, then every six months to date by the gynecologic oncologist and medical oncologist) there was no recurrence of the disease seen on either physical examination or CT imaging.

## Discussion

Our patient was diagnosed as having at least clinical stage IB2 cervical cancer at the time of presentation. From the results shown in Table 1, it seems that PNET of the cervix can happen at any age. In a literature review performed by Snijders-Keilholz *et al*. [9], the range of age at diagnosis of the disease was between 21 and 60 years. PNET of the uterine cervix belongs to the Ewing's sarcoma family [10]. Although such tumors usually occur in younger adults, often around the shoulder or hip, they can arise at many other sites including the uterine cervix. Cytogenetic studies now indicate that Ewing's sarcoma and PNET have similar histological features and should be considered as one entity [10]. In our patient's case, we did not have the facilities to test for the t(11;22) translocation found in Ewing/PNET. However, strong membranous staining with CD99 was very characteristic and sensitive. Chromogranin and synaptophysin test results were also positive. Furthermore, negative staining for a wide variety of other markers including CD3, TdT, desmin, LCA, neurofilament, CD10, CD20, cytokeratin, and CEA excluded other types of small, blue, round cell tumor that entered the differential diagnosis.

**Table 1:** Management of nine patients with primitive neuroectodermal tumor (PNET) of the cervix, as published in the English literature (1987 to 2008)

First author, year, reference	Age, gravida/para	Clinical stage/metastasis (met)	Treatment protocol	Follow-up
Russin, 1987 [1]	60 years, 2/2	IB2/no met	Internal and external RT. Residual carcinoma in endocervical curettement reason for TAH+BSO+staging laparotomy. Tumor in endocervix and implant in cul-de-sac, reason for six courses of CHT. VAC.	Alive 16 months after diagnosis
Sato, 1996 [2]	44 years, 4/2	IB2/no met on whole body X-ray and bone scan negative	TAH+LSO+pelvic lymph node dissection (all N neg), followed by unknown no. of courses of cisplatin, etoposide, adriamycin and cyclophosphamide. Second-look operation after 6 months revealed no tumor.	Alive 6 months after first operation
Horn, 1997 [3]	26 years, 3/2	IB1/no met after hysterectomy	TAH+BSO+pelvic lymphadenectomy (lymphangiosis but no met) followed by RT of the pelvis 3 years later, pulmonary met: CHT (5 FU/cisplatin)+thorax RT.	Died 4.2 years after surgery, due to met
Cenacchi, 1998 [4]	36 years	IB2/no met on whole body CT after TAH	TAH without BSO	Alive NED 18 months after surgery
Pauwels, 2000 [5]	45 years	IB2/no met	TAH (no tumor outside the cervix) followed by pelvic RT	Alive 42 months after surgery
Tsao, 2001 [6]	24 years, 3/2 pregnant	No bony lesions and no lymph node involvement	Pre-operative CHT due to large primary lesion: two alternating cycles of VAC and IE, followed by TAH+transposition of the ovaries and para-aortic LN sampling, followed by two alternating cycles of VAC and IE, and then pelvic RT.	No details of survival
Malpica, 2002 [7]	35 years	IB1/no met	TAH+BSO+selective para-aortic and bilateral pelvic lymphadenectomy followed by adjuvant CHT	Alive 5 months after diagnosis
Malpica, 2002 [7]	51 years	IB2/no met	Same treatment as the previous patient	Alive 18 months after diagnosis
Snijders-Keilholz, 2005 [9]	21 years, nulligravid	-/no met	Six courses of DIME followed by hysterectomy without adnexectomy and without lymphadenectomy followed by 5 courses of VIA	Alive 27 months after diagnosis
Goda, 2007 [8]	19 years, nulligravid	-/no met	Combination CHT with VAC, planned for further consolidation CHT after RT	Alive

CHT = chemotherapy; CT = computed tomography; DIME = doxorubicin, Ifosfamide, mesna, etoposide; IE = ifosfamide, etoposide; N neg = lymph node negative; NED = no evidence of disease; RT = radiotherapy; TAH+BSO = total abdominal hysterectomy, bilateral salpingo-oophorectomy; VAC = vincristine, adriamycin, cyclophosphamide; VID = vincristine, ifosfamide, dactinomycin.

Ewing's sarcoma must be considered as a systemic disease without adequate treatment in which more than 90% of patients die from secondary hematogenous metastases, occurring mainly in the lung. Therefore, the five-year survival rate can increase to 55% to 60% with dose-intensive cytotoxic treatment regimens in localized disease; the three-year disease-free survival rate was reported to be 15% to 22% among patients with detectable metastases at time of diagnosis [11].

As shown in Table 1, in earlier reports the approach to treatment of uterine cervix PNET was optimal local surgical treatment followed by additional treatment such as irradiation and/or chemotherapy. Recently however, neoadjuvant chemotherapy followed by local treatment has been favored because of improved results from the addition of systemic therapy. Optimal management must be based on a GOMDT decision. As all 10 cases were at stage IB and mostly with no metastasis at the time of diagnosis, it is assumed that a tumor in this location produces symptoms at an early stage. Although, as can be seen from Table 1, one patient as well as our patient underwent chemotherapy first because the tumor was too large to perform initial radical surgery, the other cases had primary local treatment mainly consisting of primary surgery (except in one patient which was first treated with radiotherapy) followed by additional local radiotherapy or systemic treatment. Metastatic disease developed after three years in one case, and this patient died after a year.

It might be considered that neoadjuvant chemotherapy is an overtreatment for the management of PNET. However, as Snijders-Keilholz *et al*. [9] mention, up to now duration of follow-up of the reported cases is too short (five to 42 months) to prove or reject this discussion. As with Ewing's sarcoma of other sites, we showed that neoadjuvant chemotherapy in metastatic cases could change an inoperable presentation to an operable state for successful local and/or regional treatment.

## Conclusions

Patients with localized PNET of the cervix should be treated with dose-intensive neoadjuvant chemotherapy followed by local treatment, and chemotherapy thereafter. Our patient was treated with a multimodal therapy regime resulting in a disease-free state for at least four years after diagnosis.

## Consent

Written informed consent was obtained from the patient for publication of this case report and any accompanying images. A copy of the written consent is available for review by the Editor-in-Chief of this journal.

## Competing interests

The authors declare that they have no competing interests.

## Authors' contributions

FR analyzed and interpreted the data regarding the histological examination of our patient. HR performed the medical treatment, and was a major contributor to writing the manuscript. FF and PT performed the surgical treatment and the follow-up. All authors read and approved the final manuscript.
